# Rare Cause for a Fluorodeoxyglucose (18F) avid Paravertebral Mass: Mediastinal Hibernoma

**DOI:** 10.1016/j.atssr.2025.10.017

**Published:** 2025-11-19

**Authors:** Akshat Saxena, Jonathon B. Ryan, Peter Grant

**Affiliations:** 1 Discipline of Surgery, University of New South Wales, Kensington, New South Wales, Australia; 2 Department of Cardiothoracic Surgery, Prince of Wales Hospital, Randwick, New South Wales, Australia

## Abstract

There is a wide range of differential diagnoses for paravertebral masses due to complex regional anatomy of the region. Positron emission tomography can help distinguish between benign and malignant lesions; malignant lesions usually have higher 18F fluorodeoxyglucose uptake. We report the case of a 70-year-old woman with a rare benign fluorodeoxyglucose–positron emission tomography avid lesion: mediastinal hibernoma.

The paravertebral region is complex and a comprehensive imaging approach is crucial for accurate diagnosis and management of tumors. Radionuclide imaging with positron emission tomography (PET) is widely used to help distinguish between benign and malignant lesions. Malignancies generally exhibit higher 18F fluorodeoxyglucose (FDG) uptake because increased glucose metabolism is a common feature of rapidly growing cancer cells. Hibernomas are rare benign neoplasms with similar differentiation to brown fat that are also FDG-PET avid and may mimic malignancy. They represent a diagnostic and management challenge.

A 70-year-old woman presented to her local medical officer with a 3-month history of left upper quadrant pain. She had a history of osteoarthritis, obesity, and previously excised benign breast lesion. A computed tomography (CT) abdomen scan was arranged, demonstrating uncomplicated diverticulitis and a left-sided paravertebral mass superior to the diaphragm. A subsequent contrast CT chest scan confirmed a 59 x 25 x 49 mm mass extending from T8 to T10 with intermixed hazy soft tissue and fat components (Figure 1). There were an enhancing component and prominent feeding/draining vessels. The mass was extrapleural and did not invade surrounding structures or extend into the neural foramina. There was no evidence of disease elsewhere. The impression was a lipomatous tumor or an atypical myelolipoma. After discussion at a multidisciplinary team (MDT) meeting a FDG-PET/CT scan was performed. The mass demonstrated intense abnormal FDG accumulation with a maximum standardized uptake value of 13.8 (Figure 2). The most metabolically active component of the mass was the medial aspect, inferior to the descending aorta. There was no pathologic uptake elsewhere. The intense metabolic activity raised high suspicion for a malignancy, but this seemed incongruous with the uniform low-density appearances on the CT scan. A biopsy of the mass was considered too risky because of its intense vascularity and close proximity to the aorta. The MDT consensus was for surgical extirpation given the diagnostic uncertainty. A thoracoscopic-guided core needle biopsy was considered but a definitive excision was preferred as it was it would complete analysis of the resected sample. The procedure was performed via a left sixth intercostal space posterolateral minithoracotomy; a thoracoscopic approach was abandoned because of the large and numerous arterial vessels supplying the mass. A firm encapsulated extrapleural mass was seen in the paravertebral gutter adjacent to the aorta and immediately superior to the diaphragm. The mass was supplied by large vessels arising from the aorta and intercostal arteries.

**Figure 1:**
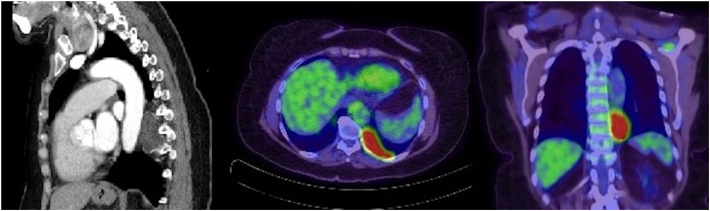
(Left) Sagittal contrast enhanced computed tomography chest scan demonstrating a paravertebral mass with prominent feeding vessels. (Middle, Right) Axial and Coronal images of a fused positron emission tomography/computed tomography scan demonstrating prominent 18F fluorodeoxyglucose uptake by the mass (maximum standardized uptake value, 13.8).

**Figure 2:**
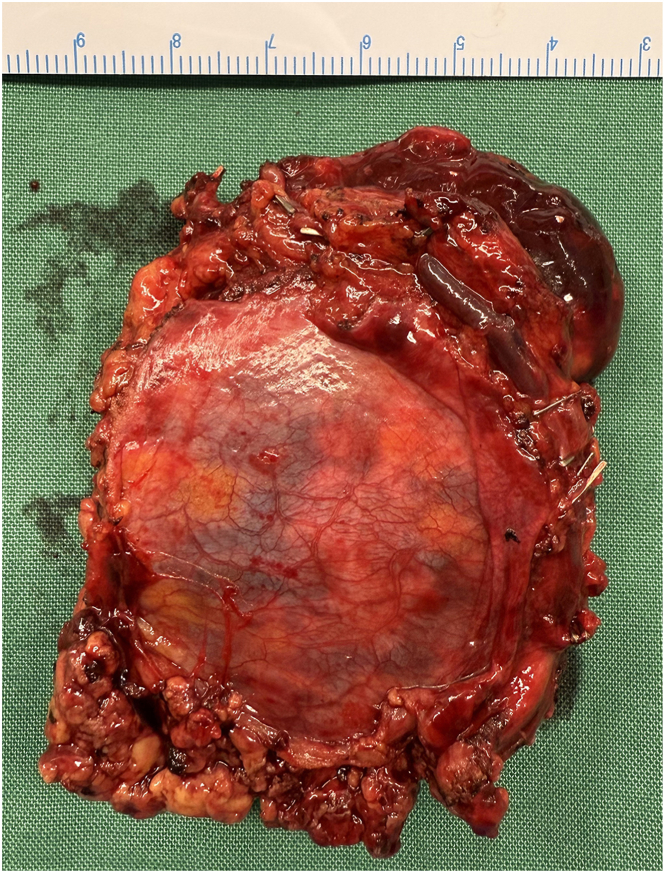
Resected paravertebral mass with scale.

First, the parietal pleura overlying the descending aorta adjacent to the mass was incised vertically. The mass was then mobilized from the aorta with the feeding vessels divided. Next, the peripheral margins were mobilized with the additional feeding vessels in the intercostal spaces being divided as they were encountered. Prominent venous branches to the hemiazygos system were similarly divided. The tumor was removed en bloc. The thoracotomy wound was closed, the patient extubated and transferred to the ward. Her hospital stay was uneventful and she were discharged on postoperative day 4. Postoperatively, histology sections demonstrated tumor consisting almost exclusively of brown fat cells consistent with hibernoma (Figure 3). There was no evidence of malignancy.

**Figure 3:**
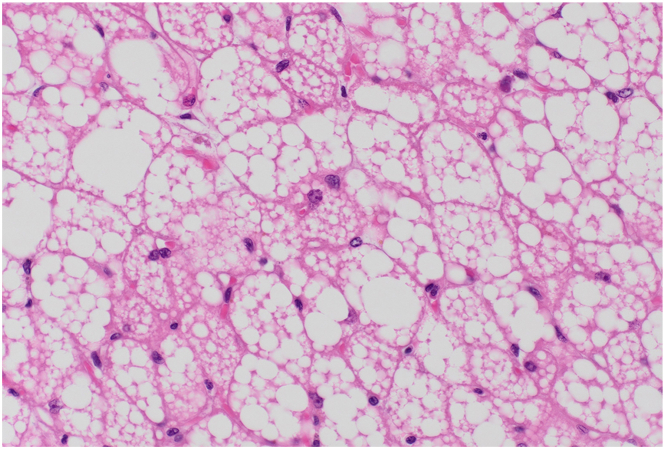
Hematoxylin and eosin stain of resected sample demonstrating multivacuolated cells with small bland nuclei and univacuolated adipocytes consistent with hibernoma.

## Comment

Hibernomas are rare benign adipocytic neoplasms that differentiate similar to brown fat.^1^ They are most prevalent in young adults with the most common sites being the thigh, shoulder, and back.^1^ Paravertebral hibernomas are exceptionally rare. Our case highlights a differential to consider when assessing a FDG avid paravertebral mass. Imaging is critical in formulating a hypothesis regarding the etiology of paravertebral masses. Due to the complex regional anatomy of the region, there is a wide range of differential diagnoses. Pathologies can arise from paraspinal soft tissue (muscles, spinal nerves, sympathetic chains, arterial/venous systems) or the bony vertebrae.^2^ Most commonly, paravertebral masses are benign neoplasms including lipomas, fibroelastic tumors, and peripheral nerve sheath tumors. Less common are malignant neoplasms, including liposarcomas and undifferentiated sarcomas or metastases. Higher FDG uptake on PET/CT is usually consistent with a malignancy. In this case, however, the intense maximum standardized uptake value reflected a benign tumor. This reflects the unique property of the brown fat that constitutes a hibernoma. Brown fat differs in function and morphology from white fat. White fat serves as an insulator and for energy storage, whereas brown fat generates heat in response to cold exposure. Brown fat is also highly vascularized with rich sympathetic adrenergic innervation, which leads to an influx of glucose via overexpression of glucose transporters. This manifests as high FDG uptake. The physiologic similarity between brown fat and malignancy can lead to hibernomas mimicking malignancy on PET/CT, as was the case in this patient.^3^ This represents a diagnostic dilemma given that the imaging appearance of hibernomas closely resembles that of connective tissue–derived malignant neoplasms (liposarcoma, atypical lipomatous tumor, high-grade rhabdomyosarcoma or lymphoma). Fortunately, hibernomas are benign, with little propensity to recur after complete excision. There have been reports where authors have attempted to elucidate the pathophysiologic mechanisms underlying the FDG-PET uptake to discriminate brown fat/hibernoma from malignancy. In one report, the authors evaluated whether the intense glucose uptake was related to adrenergic activation by repeating PET/CT scanning under full dose beta-blocker therapy.^4^ A repeated study under propranolol treatment (120 mg per day, starting 5 days before the exam) documented the virtual disappearance of FDG uptake. In our case, the MDT felt that the optimal approach was surgical excision, given the need for definitive pathologic diagnosis and the potential for mass effect with growth. A key learning point of this case for the MDT was to lower the threshold for biopsy in patients with a suspected nonmalignant FDG-PET avid mass and to consider beta-blocker suppression therapy if hibernoma is suspected. Our case report identified an unusual cause for a FDG avid paravertebral mass and highlighted the diagnostic dilemma associated in its management.
